# Femoral Osteochondritis Dissecans and Tibial Osteochondral Defect in an Adult Revealed by Bone SPECT/CT

**DOI:** 10.3390/diagnostics16111630

**Published:** 2026-05-26

**Authors:** Tzyy-Ling Chuang, Keng-Chang Liu, Chih-Wen Lin, Chun-Hsi Huang, Yuh-Feng Wang

**Affiliations:** 1 Department of Nuclear Medicine, Dalin Tzu Chi Hospital, Buddhist Tzu Chi Medical Foundation, Chiayi 622401, Taiwan; b8601139@tmu.edu.tw; 2 School of Medicine, Tzu Chi University, Hualien 970374, Taiwan; dalinchiayi@gmail.com; 3 Department of Orthopedics, Dalin Tzu Chi Hospital, Buddhist Tzu Chi Medical Foundation, Chiayi 622401, Taiwan; kengchangliu@gmail.com; 4 Endoscopic and Minimally Invasive Spine Center, Dalin Tzu Chi Hospital, Buddhist Tzu Chi Medical Foundation, Chiayi 622401, Taiwan; 5 Department of Medical Imaging, Dalin Tzu Chi Hospital, Buddhist Tzu Chi Medical Foundation, Chiayi 622401, Taiwan; 6 Department of Nuclear Medicine, Taipei Veterans General Hospital, Taipei 112201, Taiwan; 7 Department of Biomedical Imaging and Radiological Sciences, National Yang Ming Chiao Tung University, Taipei 112304, Taiwan; 8 Department of Medical Imaging and Radiological Technology, Yuanpei University of Medical Technology, Hsinchu 300102, Taiwan

**Keywords:** bone scan, osteochondral defect, osteochondritis dissecans, SPECT/CT

## Abstract

A 46-year-old woman presented with persistent right knee pain and swelling six months after a fall. MRI initially showed a lateral meniscus tear, leading to meniscus repair and later meniscectomy, but symptoms persisted. Retrospective review of the MRI revealed edema in the tibial plateau and distal femoral condyle. Arthroscopic debridement demonstrated severe synovitis, marked cartilage loss of the lateral femoral condyle with a loose body, and tibial plateau cartilage damage. Bone SPECT/CT showed bony destruction, cleft formation, and focal tracer uptake in the distal femur and proximal tibia. Femoral osteochondritis dissecans and a tibial osteochondral defect were diagnosed based on arthroscopic and imaging findings.

**Figure 1:**
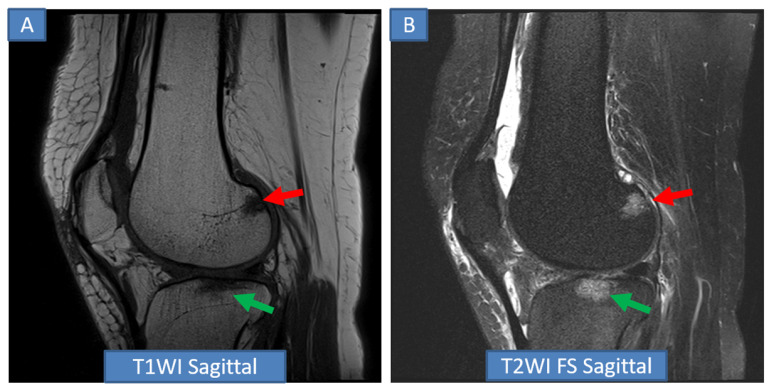
A 46-year-old woman presented with persistent right knee swelling and pain six months after a fall. Initial MRI showed a lateral meniscus tear, which was treated with repair and later meniscectomy with incomplete symptom relief. Physical examination showed swelling extending from the lateral knee to the anterior thigh, with limited extension but no tenderness. Retrospective review of MRI demonstrated edema of the right distal femoral condyle (red arrow) and tibial plateau (green arrow) with osteochondral lesions, which is low signal intensity on the T1W image (**A**) and high signal intensity on the T2W FS image (**B**).

**Figure 2:**
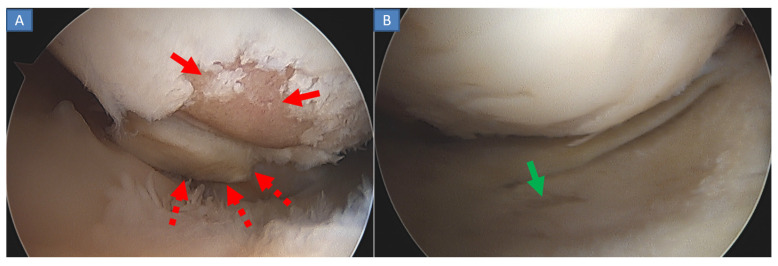
Despite Evenity treatment and repeated arthrocentesis, symptoms persisted, and cultures remained negative. Arthroscopic debridement revealed severe synovitis, >50% cartilage erosion of the lateral femoral condyle ((**A**), red arrows) with a loose body ((**A**), dashed arrows), and tibial plateau cartilage damage ((**B**), green arrow).

**Figure 3:**
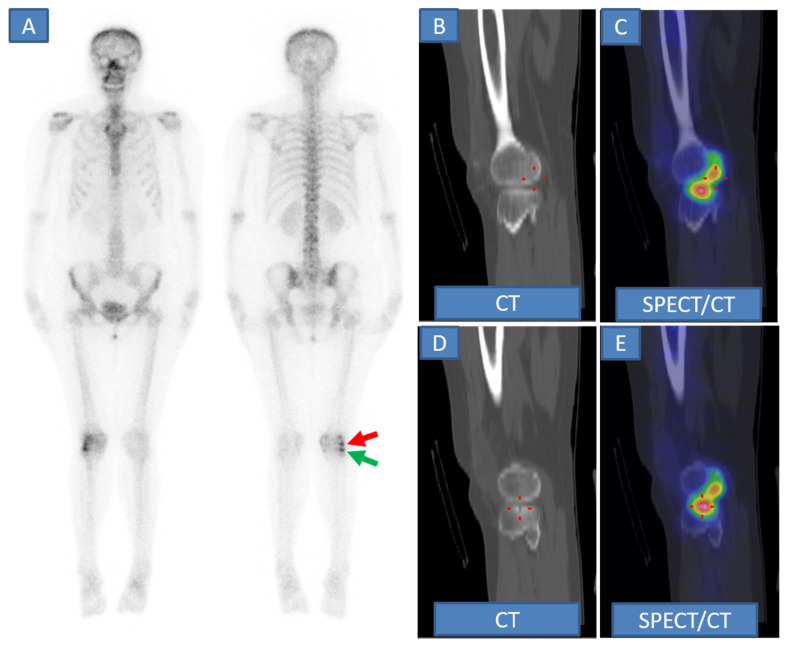
Bone SPECT/CT showed bony destruction, cleft formation, and focal radiotracer uptake in the distal femur ((**A**), red arrow; (**B**,**C**), crosshairs) and proximal tibia ((**A**), green arrow; (**D**,**E**), crosshairs), SPECT/CT with rainbow color scale. Combined imaging and arthroscopic findings supported femoral osteochondritis dissecans (OCDi) and a tibial osteochondral defect (OCDe). Cartilage lesions may occur alone or with subchondral bone involvement, the latter termed OCDe [1]. Osteochondral lesions (OCL) represent focal abnormalities of articular cartilage, the subchondral plate, and marrow, and arise from trauma, subchondral insufficiency fractures, avascular necrosis, osteoarthritis-related collapse, or OCDi [1,2,3]. OCDe commonly results from acute injury or repetitive microtrauma leading to microtrabecular fractures and impaired remodeling [3]. Early lesions typically show marrow edema, subtle cortical disruption, or hypointensity beneath the subchondral plate on MRI [2,4], whereas advanced lesions may develop contour deformity, instability, or loose bodies, increasing the risk of early osteoarthritis [3]. Although both OCDe and OCDi involve the osteochondral unit, they remain distinct. OCDe reflect localized structural loss of cartilage and subchondral bone from acute or chronic mechanical injury without a primary vascular mechanism [4]. In contrast, OCDi results from subchondral ischemia causing necrosis and potential osteochondral fragment (loose body) separation [1,5]. OCDi occurs more often in adolescents due to the vulnerability of the immature epiphysis [6]. Clinically, the acronym “OCD” is often misused to describe both conditions; however, it should strictly denote osteochondritis dissecans [2]. In this report, we use the term “OCDi” to clearly distinguish osteochondritis dissecans from OCDe, a specific subtype within the broader category of OCL. Before CT and MRI were widely used, Tc-99m phosphate scintigraphy proved valuable for detecting juvenile OCDi, owing to its sensitivity to lesion activity [7]. Bone scintigraphy remains a useful screening tool for persistent bone pain, though planar imaging has limited localization [8]. SPECT/CT or quantitative scanning improves anatomical precision and metabolic characterization, with reported value in identifying ankle and knee OCDi lesions [5,8]. Arthroscopy is reserved for persistent symptoms and to evaluate structural lesion stability across established grading systems [9,10]. OCDi is a specific osteochondral pathology caused by vascular compromise, most commonly in the knee (especially the medial femoral condyle); adult lateral femoral condyle involvement, as in this case, is uncommon but clinically important. Bone SPECT/CT is valuable for both OCDe and OCDi, demonstrating focal uptake, clefts, and subchondral destruction while providing metabolic detail. Early and accurate diagnosis is essential for appropriate management in adults with persistent knee pain unresponsive to conventional therapy.

## Data Availability

Due to ethical restrictions, the data presented in this study are available only upon request from the corresponding author.

## Abbreviations

The following abbreviations are used in this manuscript:

CT	Computed tomography
FS	Fat suppression
MRI	Magnetic resonance imaging
OCDe	Osteochondral defect
OCDi	Osteochondritis dissecans
OCL	Osteochondral lesions
SPECT/CT	Single photon emission computed tomography/computed tomography
